# Population physiologically-based pharmacokinetic model incorporating lymphatic uptake for a subcutaneously administered pegylated peptide

**DOI:** 10.1186/s40203-016-0018-5

**Published:** 2016-03-01

**Authors:** Elliot Offman, Colin Phipps, Andrea N. Edginton

**Affiliations:** School of Pharmacy, University of Waterloo, 200 University Ave W, Waterloo, ON N2L 3G1 Canada

**Keywords:** Physiologically-based pharmacokinetic model, Subcutaneous, Peptide, Pegylated, Lymph

## Abstract

**Purpose:**

Physiologically-based pharmacokinetic (PBPK) models provide a rational mechanistic approach for predicting the time course of macromolecules in plasma. Population PBPK models for large molecules necessitate incorporation of lymphatic circulation to mechanistically account for biodistribution. Moreover, characterization of subcutaneous absorption requires consideration of the microvascular transit from the injection site to the systemic circulation. A PBPK model for a pegylated peptide conjugate, previously developed for primates, was modified to describe the lymphatic uptake in a population of humans by incorporation of interindividual variability in the lymphatic circulation and a unique lymphatic drainage compartment (LDC). The model was then used to simulate the time course of the drug in a population of humans and compared to the same drug administered to a group of human subjects participating in a first-in-human study.

**Methods:**

Organ, blood and lymph masses for the population were sampled from either normal or log-normal distributions. Blood flows were calculated for each organ based on mean organ perfusion per gram of organ tissue and lymphatic flow was set as a fixed fraction of blood flow. Interindividual variability in lymphatic volume was assumed to be similar to that of blood volume. The volume of the LDC was parameterzed as a fraction of the injection volume. Sensitivity analysis was performed to study uncertain parameters and distribution assumptions.

**Results:**

The population generator was capable of simulating a virtual population incorporating the lymphatic circulation. Incorporation of a LDC resulted in similar line shape relative to the observed data and incorporation of anthropometric variability accounted for individual differences in the absorption and elimination phases across all dose cohorts. Line shape was sensitive to the inclusion of LDC while peak and elimination portions of the time course were influenced by the magnitude of variance assumed for blood volume and renal clearance, respectively.

**Conclusion:**

Lymphatic circulation can be incorporated into a population PBPK model assuming similar interindividual variability as observed for blood volume. Incorporation of an LDC, where the volume of this transit compartment is proportional to the SC injection volume may be an important mechanistic means of predicting the transit from the SC depot to the systemic circulation.

**Electronic supplementary material:**

The online version of this article (doi:10.1186/s40203-016-0018-5) contains supplementary material, which is available to authorized users.

## Background

The importance of the subcutaneous (SC) route to the drug development industry is evident in the growing number of drug products available for SC administration. In 2014, the U.S. Food & Drug Administration approved 41 new molecular entities and biological licensing applications, four of which had first approvals for subcutaneous (SC) administration (2015). The industry has also witnessed conversion of intravenous (IV) to SC routes for a number of therapies where treatment that was previously relegated to a hospital can now largely be addressed in an ambulatory setting (e.g. IV heparin to SC low molecular weight heparins for treatment of deep vein thrombosis). With a growing emphasis on SC administration as the primary route for development, reliance on reliable pharmacokinetic (PK) scaling methods (pre-clinical animal species to human) is correspondingly increasing.

When designing clinical pharmacology (Phase 1) and in particular first-in-human (FIH) studies, there is considerable importance placed on predicting the peak (Cmax) and overall exposure (AUC). This is particularly critical when relating the exposure observed in pre-clinical species to observed adverse events. Appreciating the temporal relationship between dosing time and Cmax, as well as an estimate of the drug’s plasma half-life (T1/2), are useful for *a priori* design of early human research studies to mitigate the need for protocol amendments and reduce the burden of interim PK assessments.

In traditional allometric theory, prediction of human PK from animals has largely focused on estimating mean human exposure by scaling the mean clearance value of three or more preclinical species (Mahmood and Balian 1996; Mordenti 1986). For macromolecules, and particularly for monoclonal antibodies (mAbs) exhibiting linear PK, single-species (primates) simplified allometric techniques have yielded useful predictions (Offman and Edginton 2013; Wang and Prueksaritanont 2010; Deng, et al. 2011; Dong, et al. 2011; Ling, et al. 2009). These methods however, provide no information as to the time course of a drug after administration, and have principally been employed for the IV route of administration. Dedrick plots have been demonstrated to have some utility in predicting the time course of IV administered monoclonal antibodies (mAbs), but have not yet been reported to yield similar predictive capacity for SC administered proteins (Ling, et al. 2009).

Predicting the SC time course of a drug in a population of humans from animal models is considerably more complex compared to prediction for a “mean human”. For macromolecules, this is further complicated by a lack of knowledge regarding extravascular bioavailability and/or non-linearity in PK.

Whole body physiologically-based PK (WB-PBPK) modeling provides a rational mechanistic approach for predicting the time course of drugs in the vasculature and potentially other tissue compartments, and has demonstrated utility in describing the mean time course of IV administered macromolecules in both pre-clinical and human species (Baxter, et al. 1995; Ferl, et al. 2005; Davda, et al. 2008; Urva, et al. 2010; Shah and Betts 2012). To extrapolate to SC administration however, these models may require modification to better account for local lymphatic drainage from the SC depot site.

The lymphatic system provides unidirectional transport for fluid and protein by collecting constituents from the interstitial space and returning them to the blood (Swartz 2001). While transport is convective in nature, movement of lymphatic fluid and macromolecules transported into and by the lymphatics can be influenced by muscle contractions and ambient temperature (Olszewski, et al. 1977; O’Morchoe, et al. 1984; Swartz 2001). Lymphatic vessels are categorized into capillaries, larger collecting vessels, nodes, trunks and ducts, and likely, many capillaries drain a single injection site (Porter, et al. 2001; Swartz 2001). Cannulation techniques in animal models have allowed for the quantitation of macromolecule uptake into the lymphatics, and whilst seminal in confirming lymphatic involvement in biodistribution and contributing to our understanding of how macromolecule size influences uptake, they do not address the actual local drainage dynamics from the depot into the immediate lymphatic capillaries (Porter, et al. 2001; Supersaxo, et al. 1990; Supersaxo, et al. 1988). Skin lymphatics include superficially spread, subpapillary fine mesh and deeper vessels which empty into larger vessels draining the SC space before reaching the collecting ducts (Lubach, et al. 1996). Mathematical modeling lymphatic drainage remains challenging, as there is no non-invasive method for differentiating the volume of lymphatics attributed to local drainage at the site of drug administration and that attributed to the remainder of the lymphatic vessels.

WB-PBPK models for macromolecules and particularly mAbs have universally incorporated a lymph node compartment with convective transport dragging drug across the organ vascular-interstitial interface, into the lymph node compartment and subsequently into venous circulation (Baxter, et al. 1995; Urva, et al. 2010; Ferl, et al. 2005; Shah and Betts 2012; Davda, et al. 2008; Offman and Edginton 2015). Although mean values for a lymph node compartment are reported, it is unclear as to whether the reported volumes relate to the sum of all nodes, ducts, collectors and capillaries or to what extent these vary among individuals. Furthermore, population PBPK algorithms, available in the public domain, have not reported compartment characteristics (i.e. mean, variance) for the lymphatic system or lymph flow, and consequently don’t readily lend themselves to the study of SC administration of macromolecules in a population of individuals (McNally, et al. 2014; Willmann, et al. 2007).

Employing a WB-PBPK modeling approach, we previously characterized the SC time course of a novel pegylated peptide conjugate in primates which was then scaled to humans at three different dose levels in a FIH single ascending dose (SAD) trial (Offman and Edginton 2015). Upon further examination across a wider range of doses, the model predictions suggested further model refinement in the lymphatic uptake processes would improve predictive capacity of the model, particularly in the very early portion of the concentration vs. time profile.

To further investigate the contribution of local lymphatic capillary drainage from the SC space on the time course and shape of the plasma concentration vs. time profile (CPT), and to improve our understanding as to which parameters influence the variability of the CPT in a population, we endeavored to expand on our previous work and develop a population PBPK model which incorporates both lymphatic drainage and overall lymphatic system compartments.

## Methods

### Observed datasets

CPT data was obtained for a novel pegylated peptide conjugate (approximately 45 kDa) as it transitioned from preclinical to early clinical development. The compound (name and target withheld for commercial proprietary purposes), predominantly consists of a freely water soluble, linear PEG-40 conjugated to a small (approximately 1 kDa) peptide portion. The current evaluation includes concentration and anthropometric data from 20 healthy Australian male subjects, 18–55 years of age and within a weight range of 60–80 kg who participated in a FIH, SAD investigation. The investigation was conducted under good clinical practice and according to the ethical principles outlined in the Declaration of Helsinki. Each subject received a single SC dose between 45 mg and 720 mg into the abdominal region with sequential PK sampling post-dose until approximately 1050 h. The concentration of the injection ranged from 100 mg/mL to 150 mg/mL with multiple injections for some dose levels such that the volume in any single injection would not exceed 2 mL. Plasma was analyzed employing an LC-MS/MS method with a limit of quantitation of 1 ug/mL.

### Model structure

**Fig. 1:**
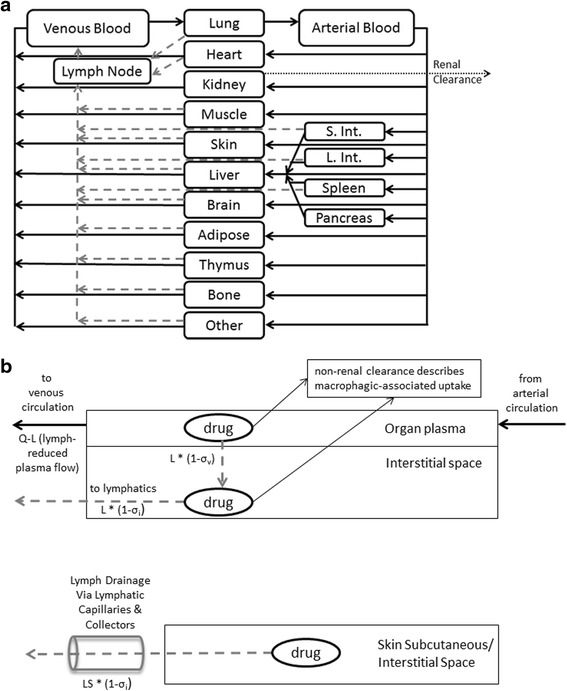
**a** Structure of a whole-body PBPK platform (adapted from Shah and Betts 2012). Solid black arrows indicate plasma flow. Dark grey dashed arrows indicate lymphatic transport. S. Int. and L. Int. represent small and large intestines. Each organ compartment includes lymphatic flow emptying from the organ into the lymph nodes. For IV administration, drug is administered into the “Venous Supply”. For SC administration, drug is administered into the “Skin Compartment” interstitium. **b** Sub-compartment model for all organs other than skin (top) and skin (bottom)

The base model structure has been previously described (Offman and Edginton 2015), and was slightly modified to better characterize the SC drainage into the lymphatics. Representative mass balance equations are provided for reference in Additional file 1. The proposed overall and sub-compartment structures are based in part on the PBPK platform developed previously by Shah and Betts and is depicted graphically in Fig. 1a-b (Shah and Betts 2012). The overall structure consists of a unique compartment for each of the venous and arterial circulation, a lymph node compartment and 15 individual organs where each organ consists of a vascular and interstitial sub-compartment. The skin interstitial compartment is further sub-divided into a depot and residual space where the SC dose inputs directly into the depot. The SC depot volume was parameterized as being equivalent to the total injection volume, which varied from 0.45–4.8 mL depending on the dose level and concentration injected. Transport from the interstitium into the lymphatic space is via a convective flow with a small degree of resistance parameterized as lymph flow and a lymphatic reflection coefficient (σ_*i*_), as proposed by Garg and Balthasar (2007). In our previously developed model based on primates,, drug in the SC depot space emptied directly into a lymphatic compartment (Offman and Edginton 2015). In the current model, an intermediate anatomical volume was added in order to include the localized lymphatic drainage from the SC depot into adjacent lymph vessels, which we will refer to as the lymphatic drainage compartment (LDC). The LDC subsequently drains into the greater lymphatic system compartment. Lymphatic flow empties from the lymph compartment into the venous circulation and re-enters the interstitial space in a one-way circuit via the same lymphatic convective flow. Transfer from the organ plasma vascular space to the interstitial space is similarly driven by convective flow and constrained by vascular reflection coefficients (σ_*v*_) that were initially set at values proposed by Shah and Betts (2012). However, as per the previous model, all organ vascular reflection coefficients are scaled by the parameter σ_*sf*_, which was previously optimized (Offman and Edginton 2015). Fraction vascular (Fvv) and interstitial (Fvic) were retained at the same values as in the original model (Offman and Edginton 2015).

### Virtual population development

Prior to simulation of a virtual population, the target population for simulation needs to be defined. Since the observed PK data was obtained in a group of Caucasian Australian men, average height and weight and corresponding coefficients of variations for these anthropometric measurements in non-obese Australian males were obtained from Craig, et al. (2001). As described by Willmann et al., first, height values were randomly drawn from a normal distribution for a population of 1000 male subjects followed by sampling of each organ compartment mass for each individual in the virtual population (Willmann, et al. 2007). To mitigate the likelihood of individuals with the same height being allocated identical organ weights, organ masses were drawn either from a normal or log-normal distribution (Table 1) that was re-centered to the size of the individual employing an allometric scaling factor according to the ¾ power rule (Willmann, et al. 2007). To avoid the selection of extreme outliers, organ mass distributions were symmetrically truncated to the 95^th^ percentile and, when necessary to prevent negative masses, constrained at the lower bound to one tenth the re-centered mean value. A visual check was then performed to evaluate the impact of truncation and constraining the lower bound by repeating the simulations for all dose levels in the observed data where parameters initially drawn from a normal distribution were then drawn from a log-normal distribution.

**Table 1:** Anatomical and physiologic parameters with distributions used in the population PBPK model

	Mean^a^	CV %	Distribution	CV % Source
Mean Weight	71 (kg)	/	/	/
Compartment Mass	% Body Weight	/	/	/
Adipose	18.540	0.43	Log-normal	(Heymsfield, et al. 2007)
Brain	1.968	0.10	Normal	(Heymsfield, et al. 2007)
Blood (Arterial/Venous)	8.005	0.22	Log-normal	(Feldschuh and Enson 1977)
Bone	13.952	0.14	Normal	(Heymsfield, et al. 2007)
Heart	0.448	0.19	Normal	(de la Grandmaison, et al. 2001)
Kidney	0.421	0.25	Normal	(de la Grandmaison, et al. 2001)
Large Intestines	1.52	0.2	Normal	(McNally, et al. 2014)
Liver	2.707	0.23	Normal	(de la Grandmaison, et al. 2001)
Lung	1.569	0.36	Log-normal	(de la Grandmaison, et al. 2001)
Lymph	0.359	0.22	Log-normal	Empirically assumed to be similar to circulatory system variability
Muscle	40.702	0.16	Log-normal	(Heymsfield, et al. 2007)
Other	Remainder not accounted for by other organs	0.2	Normal	Empirically selected
Pancreas	0.136	0.27	Normal	(de la Grandmaison, et al. 2001)
Skin	4.477	0.1	Log-normal	Empirically selected
Small Intestines	1.068	0.12	Normal	(McNally, et al. 2014)
Spleen	0.244	0.56	Log-Normal	(de la Grandmaison, et al. 2001)
Thymus	0.008	0.05	Log-Normal	Empirically selected
Hematocrit	0.42	0.02	Normal	(Jacob, et al. 2012)
Renal Filtration Fraction	0.20	0.0294	Normal	(Ritz, et al. 1998)
Vfrac	0.25	0.68		Fitted Parameter

^a^Mean organ mass was obtained from the BioDMET database (Graf, et al. 2012)

All model compartments, with the exception of skin, blood and lymph were scaled according to equation (1), where the mean mass of each organ *O*, denoted ${M}_O^{mean}$, is dependent on a single variable, in this case the body height of the individual, *H*_*indiv*_, via an equation of the form ${M}_O^{mean}=c{H}_{indiv}^p$, where *c* is a sex- and race-dependent constant and *p* is a chosen exponent. Organ masses for a reference individual $\left({M}_O^{ref}\right)$ were obtained from the BioDmet database where *H*_*ref*_ is the height of a reference individual weighing 71 kg with a body mass index of 24 kg/m^2^ (corresponding to a height of 172 cm) (Graf, et al. 2012). Where organ volume and or mass were to be interconverted, density values for organs were obtained from ICRP references (2002a).

1 $$ {M}_O^{mean}={M}_O^{ref}\times {\left(\frac{H_{indiv}}{H_{ref}}\right)}^{3/4} $$

Body height has been identified previously by de la Grandmaison as a better predictor of organ size in the majority of cases and the formula is rooted in allometric theory (de la Grandmaison, et al. 2001). An exponent of ¾, although previously reported, was largely empiric for the current report, and others have used a range of values, upwards of 2, in a similar fashion (Willmann, et al. 2007; Bosgra, et al. 2012).

Blood and lymph mass means were scaled based on reference body weight (*W*_*ref*_, equation 2) as blood and lymph vessels are assumed to increase with increasing body weight as opposed to height.

2 $$ {M}_O^{mean}={M}_O^{ref}\times {\left(\frac{W_{indiv}}{W_{ref}}\right)}^{3/4} $$

Skin mass was scaled based on body surface area (BSA) as per equation 3 where BSA was estimated based on equation 4 and where the constant values for *a*, *b* and *c* were as proposed by Gehan and George (*a* = 0.0235, *b* = 0.515, *c* = 0.422) (Gehan and George 1970).

3 $$ {M}_{skin}^{mean}={M}_{skin}^{ref}\left(\frac{BS{A}_{indiv}}{BS{A}_{ref}}\right) $$

4 $$ BS{A}_{indiv}=a\times {\left({W}_{indiv}\right)}^b{\left({H}_{indiv}\right)}^c $$

The total body mass (BM) of a virtual individual was then calculated as the sum of the bloodless organ masses, lymph mass, skin mass and blood mass and the BM of the final population individuals (*n* = 1000) included only individuals within the range of 60–80 kg, consistent with the observed population. As previously suggested by Peters blood mass was partitioned as 2/3 venous and 1/3 arterial (Peters 2008).

For the derivation of organ-specific blood flows in each individual, mean perfusion values were first calculated for each organ to serve as a reference value assuming that perfusion rates would be constant across the population. Organ reference perfusion values were obtained by multiplying the cardiac output in a reference 71 kg male (Graf, et al. 2012) by the blood flow fraction to that organ and then dividing the reference blood flow by the mean organ mass. Individual organ blood flows (QB) were then obtained by multiplying the organ weight of the individual by the reference perfusion value. Plasma flow (QP) was derived by multiplying QB by a factor of 1-hematocrit (hct), where hct was assumed to be log-normally distributed (Table 1).

Consistent with the PBPK model for a reference male, lymph flow was set at a constant fraction of blood flow (LO) where the previously employed value of 0.2 % was used for all organs except skin (LS), which was set to 0.1 % (Offman and Edginton 2015). The fraction of 0.2 % corresponds to the upper range of lymph flow reported by Swartz whereas 0.1 % was obtained by optimization in the reference male model (Swartz 2001).

Clearance of the pegylated protein was previously characterized by both renal (RCL) and non-renal clearance (NRCL). RCL was set at 0.1 % of glomerular filtration rate (GFR) consistent with previously investigated PEG-conjugated therapeutics. To allow for incorporation of interindividual variation in GFR, the GFR was derived from the product of renal plasma flow and filtration fraction (FF) where FF was drawn from a log-normal distribution (Table 1) (Baumann, et al. 2014; Offman and Edginton 2015). The acronym FGFR is used to represent the fraction of glomerular filtration attributed to renal clearance. To avoid the risk of including individuals with GFRs in the impaired region of glomerular function, and to avoid extremely large values for GFR, only individuals with a GFR within the range of 90–150 mL/min were used in the simulation (Delanaye, et al. 2012).

For the compound in question, NRCL was assumed to occur by both macrophagic uptake of the non-pegylated moeity and by non-specific cleavage of the pegylated chain (Caliceti and Veronese 2003). NRCL was optimized in primates in our previous model and for the current model NRCL in the primate was scaled to each simulated human based on the body weight ratio of a simulated human individual and the mean weight of the primate (3.4 kg) (Offman and Edginton 2015). The NRCL was then apportioned based on the relative volume of each compartment in which NRCL was assumed to occur.

### Optimization of LDC

As the volume of drug product injected into the SC space increased, we hypothesized that there would be a proportional spreading of drug substance within the interstitial space, as opposed to expansion, thus increasing the surface area for lymphatic drainage. The volume then attributed to the LDC was considered proportional to the volume of the SC depot compartment. LDC volume was parameterized as a fraction of the SC depot compartment, denoted as Vfrac and estimated by fitting to the dose-normalized CPT data for all observed subjects. For this procedure, the PBPK model for an average human was coded into Phoenix NLME (v1.3, Certara) and estimation of the population mean Vfrac and between subject variability (as a log-normally distributed random effect) was performed using the first-order conditional estimation with interaction algorithm (FOCE-ELS).

Estimation was performed by considering the actual volume injected since the concentration varied across the five dose cohorts where all remaining parameters were fixed at the mean values previously optimized for a virtual human (Offman and Edginton 2015). An additional sensitivity analysis was performed whereby the optimization of Vfrac was based solely on the CPT data obtained for a single dose level in the human investigation. An objective function calculated as the absolute, average deviation of the median predicted concentration vs. median observed concentration at each nominal time point for each study cohort was employed to discriminate between models with and without an LDC.

### Model qualification

All simulations were performed using MATLAB® (v2014b, Mathworks). Adequacy of the model in describing the study population was assessed following simulation of 1000 virtual male subjects based on the study population characteristics with body weight constrained to the per protocol specified body weight (60–80 kg) and normal GFR (90–150 mL/min). Histograms were generated for height, weight and body mass index (BMI) to verify the generated population was consistent with the study population and included plausible individuals.

### Sensitivity analysis

A sensitivity analysis was performed to evaluate the model response to perturbation of the mean values of the parameters deemed to be the most uncertain and to evaluate the effect of incorporating or removing a distribution on certain parameters where the distribution was deemed uncertain. Mean parameter sensitivity was performed for Vfrac, σ_*sf*_, σ_*i*_, NRCL, LS and FGFR. For each perturbation, 100 individuals were simulated at the lowest dose level (45 mg) and one parameter at a time was perturbed by ±10 %, except σ_*sf*_, which was perturbed upwards of 1 % to avoid any single organ σ_*v*_ from exceeding 1. To evaluate sensitivity, non-compartmental analysis was performed on the median (50^th^ percentile) simulated concentration vs. time profile to derive the AUC0-inf and Cmax. Change from the final model (with LDC) as a percent was expressed and plotted graphically by perturbation of the mean parameter value.

A sensitivity analysis on distributions was also performed by simulating 1000 individuals and either perturbing or removing a parameter distribution one at a time. The simulations for each scenario were plotted and compared. This analysis was completed to define the importance of specific parameter distributions on overall CPT variability.

## Results

**Fig. 2:**
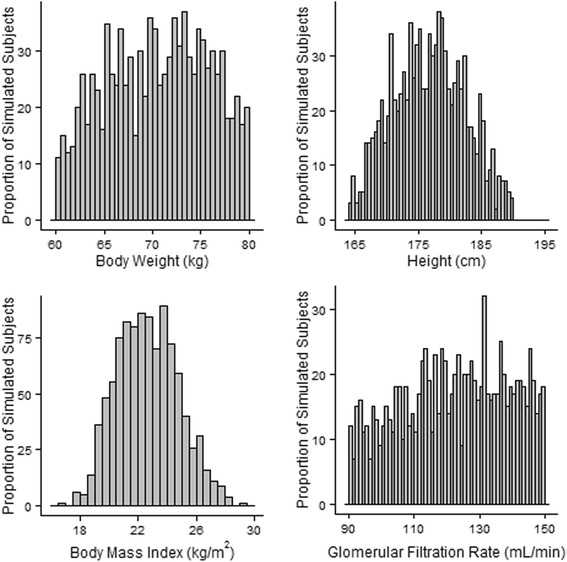
Model output anthropometric distribution of weight (upper left panel), height (upper right panel), body mass index (lower left panel) and derived glomerular filtration rate (lower right panel) for 1000 simulated individuals

One of the primary objectives of this investigation was to develop a population generator with a lymphatic system component. As a population generator, the model produced a population consistent with the study population (Fig. 2). With body height sampled from a normal distribution and total BM truncated to a range of 60–80 kg, the resultant BMIs fell largely within the typical range normally included in Phase 1 healthy volunteer research (18–28 kg/m^2^), with only a few of the 1000 simulated individuals falling outside this range. The histogram for GFR, calculated as a product of the individual FF and renal plasma flow verified that the final population included only males with normal renal function within the specified range of 90–150 mL/min.

Using the mean virtual human model and the observed dose-normalized data, Vfrac was estimated with high precision (16.8 %) with a mean and interindividual coefficient of variation of 0.25 (68 %). This value represents the volume of LDC expressed as a fraction of the SC depot volume. Although sampled from a normal distribution, Vfrac for each individual was greater than zero for simulated individuals due to the truncation and constraining methods applied to the organ mass distributions. A sensitivity analysis confirmed that similar results were obtained regardless of whether Vfrac is sampled from a normal distribution with constraints versus sampling from a log-normal distribution suggesting the type of distribution does not influence the predictive capacity of the model (Willmann, et al. 2007; McNally, et al. 2014).

**Fig. 3:**
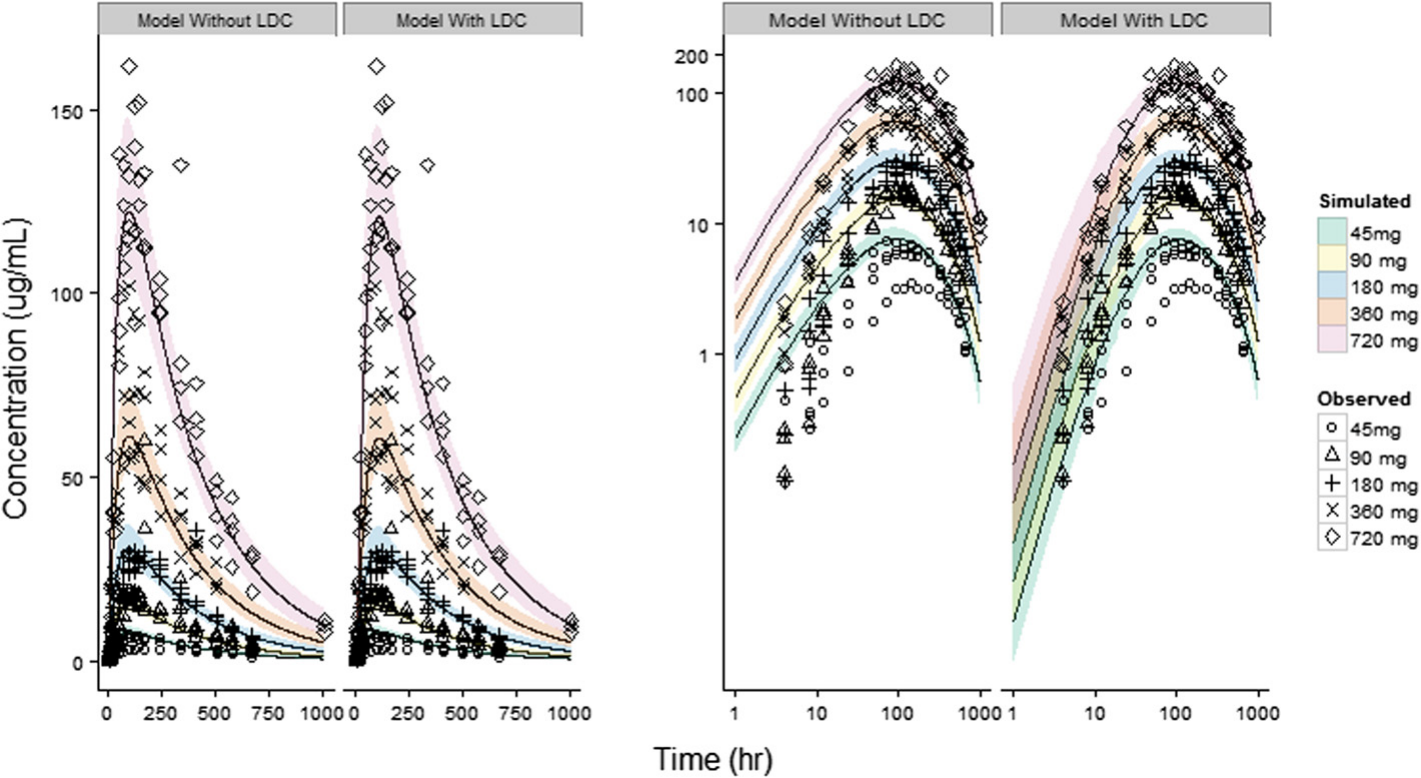
Model simulated and observed plasma concentrations of a linear PEG-40 conjugated peptide versus time in humans on linear scale (left panel) and log scale (right panel). Within each panel, the left set of profiles represents the model without a lymph transit compartment and right side, with a lymph transit compartment incorporated. Closed geometric symbols represent unique individual subjects across 5 dose levels. Solid lines and grey shaded ribbon represents the median and 5^th^–95^th^ percentile simulated concentrations from respective models

Figure 3, illustrates the comparison of the models with and without LDC when simulated for a population (*n* = 1000, 200/dose level). Visual inspection of the log-log CPT (Fig. 3, right panel) illustrates an over-prediction of the absorption phase when a LDC is not incorporated. Inclusion of LDC resulted in a reduction of the objective function from 107.11 to 21.10, relative to the model excluding LDC and improved the prediction of the absorption phase, particularly in the first 10 h post-dose.

The model, with LDC, predicted the interindividual variability in all phases of the CPT (i.e. absorption, peak and elimination) with observed data points falling on either side of the 50^th^ percentile curve and a few individual observations falling outside the 5^th^–95^th^ percentile ribbon. The one exception being a subject in the initial dose cohort (i.e. 45 mg dose) who clearly exhibited an anomalous profile, likely due to improper injection technique, as repeat analysis by the bioanalytical laboratory confirmed the measured concentration values.

**Fig. 4:**
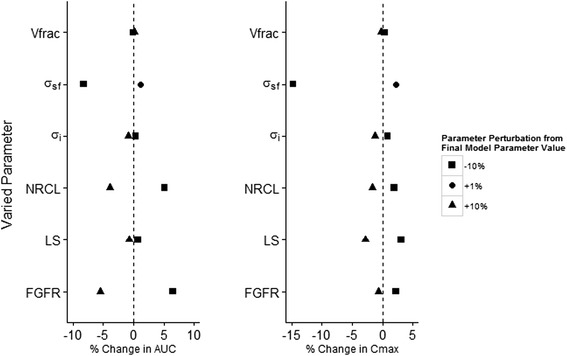
Mean parameter sensitivity perturbations vs. percent change in AUC (top left), Cmax (top right) and Tmax (bottom left) for the 50^th^ percentile following simulation of 100 subjects. Closed geometric symbols represent perturbations as indicated in the legend

Using the one-at-a-time sensitivity test for mean parameters demonstrated that in spite of a profound influence on the early portion of the time course for this particular drug, the impact of parameter perturbation on peak and overall exposure (i.e. Cmax and AUC) was relatively small (Fig. 4). The largest effect in terms of % change from the final model resulted from a -10 % perturbation of σ_*sf*_ on both Cmax and AUC. As described in the methods section, an upward perturbation was constrained to 1 % to avoid any of the individual organ vascular reflection coefficients from exceeding 1. Even so, a 1 % change in the mean vascular reflection coefficient scaling factor resulted in a visually detectable change in AUC and Cmax, confirming the model sensitivity to this parameter. Unsurprisingly, perturbations in FGFR and NRCL parameters, which influence renal and non-renal clearance, resulted in the next largest percent change in AUC, whilst having little influence on Cmax.

**Fig. 5:**
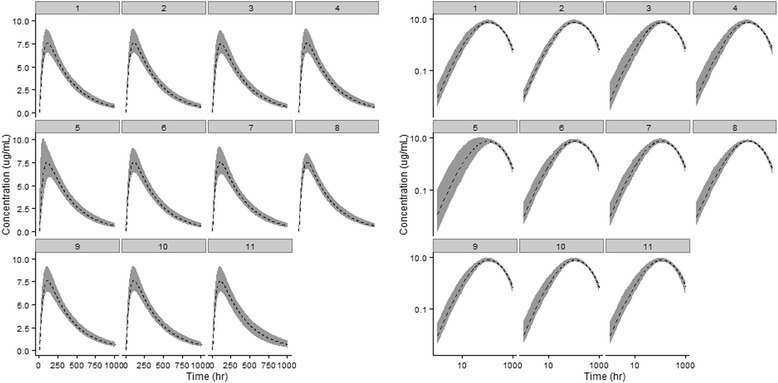
Median (dashed line) and 5^th^–95^th^ percentile (shaded ribbon) following simulation of 1000 virtual individuals on linear scale (left panel) and log scale (right panel). For sub-panels 1–11 in each panel, the following unique scenarios are presented: (1) Final model (2) 0.5-fold final model CV % for Vfrac (3) 2-fold final model CV % for Vfrac (4): Addition of 10 % CV % on LS (5) Addition of 50 % CV % on LS (6) Addition of 10 % CV % on σ_*i*_ (7) Addition of 50 % CV % on σ_*i*_ (8) Removing distribution on blood mass (9) Removing distribution on lymph mass (10) Removing distribution on skin mass (11) Addition of a 20 % CV % on FGFR

To illustrate the impact of varying parameter distribution assumptions on the three phases of the CPT, scenarios are presented on both linear and log scales (Fig. 5). Of the parameters tested, applying a distribution assumption to LS, the parameter defining skin lymphatic flow as a fraction of skin blood flow, had the greatest influence on the interindividual variation in the absorption phase (Fig. 5 panels 4 & 5). Whereas in the model development, only a mean valuefor LS was assumed, coefficients of variation of 10 and 50 % were empirically selected and the width of the 5^th^–95^th^ percentile ribbon expanded as the variability increased. In addition to LS, variability assumptions for the Vfrac parameter also influenced the interindividual variability in the absorption phase of the CPT. Halving and doubling the estimated variability assumption from 68 to 34 % and 136 %, respectively resulted in a corresponding narrowing and widening of the simulated values supporting our hypothesis that the variability assumption for this parameter is important in the prediction an SC administered pegylated macromolecule CPT (Fig. 5, panels 2 & 3). With respect to the peak portion of the CPT, variation in LS and blood volume appeared to be the most influential parameters when the distributional assumption was perturbed. For blood volume, the perturbation scenario included removing the variability in its entirety for this parameter and simulating a population based only on the mean blood volume value (Fig. 5, panel 8). This change considerably narrowed the interindividual variability at the peak of the curve which leads to an imprecise prediction of Cmax, thus supporting the importance of including the selected blood volume variability employed in the final model. Adding distributional assumptions to FGFR, which was assumed to be static for the final model simulations at 0.1 % of GFR, resulted in an obvious broadening of the interindividual variability in the latter portion (i.e. elimination) of the CPT (Fig. 5, panel 11). Of the other parameters tested, none had obvious visually detectable effects across the stages of the concentration vs. time course.

## Discussion

The compound employed in the current evaluation and the base structural PBPK model were the same used in our previous publication illustrating the utility of incorporating a SC depot in the prediction of a human CPT directly from a single non-human primate species (Offman and Edginton 2015). The molecule’s biodistributional properties were assumed to be largely attributable to the pegylated moiety, which represents >96 % of the molecular weight. The current *in silico* investigation demonstrates the scale-up from monkeys directly to a population of humans across a range of body sizes as opposed to a virtual human (weighing 70 kg).

The major objectives of this evaluation were two-fold and inter-related. The first being to develop a population PBPK model which incorporates distributional assumptions for the lymphatic system which could potentially be applied to large molecule PK predictions in a human population. The drug independent aspects of the model, being mechanistic in nature, can thus be applied to generating a population of individuals regardless of weight and height range and thus can be used to estimate the variability in a more diverse population than what was studied in the current report.

Conversion of the model to a population model was non-trivial as there is a paucity of literature to support integrating lymphatic anthropometric variability into PBPK models. Initially, we followed previous authors in terms of the distribution type (i.e. normal vs. log-normal) and, where possible, employed similar variability estimates (Table 1) (Willmann, et al. 2007; McNally, et al. 2014). For some organ compartments however, either there was no information to inform said distribution or the variability appeared unusually high. It may seem counterintuitive to employ normal distributions with respect to organ sizes given the possibility of drawing implausibly small or even negative values. However, to be consistent with reported literature we elected to retain distributions as previously reported. Instead we performed an informal sensitivity analysis which concluded no appreciable difference in the prediction interval across the simulated dose cohorts regardless of whether a distribution was log-normal or not. In our final model we employed two tiers of safeguards to mitigate the risk of drawing extreme outliers or negative values when the distribution was set as normal, with the first being truncation to the 95^th^ percentile of the distribution and the second, constraining the lower bound to one tenth the mean value. Intuitively, where future data supports, population simulations should consider log-normally distributions to avoid the likelihood of negative or uncharacteristically low values being drawn from the distribution.

Prior to truncation of GFR, values within the initial population led to values interpreted as renally impaired or unusually high. After a thorough investigation, we confirmed that kidney perfusion and renal plasma flow on a per gram of tissue basis were consistently within the expected range and consequently the root cause was attributed to a larger than likely coefficient of variation for male kidney mass used as an input into the model (McNally, et al. 2014; Willmann, et al. 2007; de la Grandmaison, et al. 2001). As a further safeguard against inflated variability in kidney mass, especially given the importance of renal elimination to the drug in question, the model was set to reject any virtual individuals with GFRs less than 90 mL/min, due to being associated with renal impairment, and above 150 mL/min range, which normal healthy individuals rarely achieve, even when normalized to 1.73 kg/m^2^ BSA (2002b). This exercise suggests that reported values for kidney mass variability may necessitate further evaluation to ensure kidney function is appropriately simulated in the virtual population.

As described in the Methods section, we empirically assumed that the variability in lymphatic system volume would be equal to that of the variability in blood volume. Although there is not specific data to inform this hypothesis, lymphatic vessels represent a parallel circulatory system and thus an assumption of blood volume based on individual size was deemed a reasonable approach.

The second objective of this *in silico* investigation was to explore the advantage of incorporating a LDC to better represent local drainage from a SC depot space, to facilitate predictions of SC administered macromolecules.

Lymph collector vessels which run through the SC space picking up macromolecules, are reported to not reach the deep fascia until a node is reached, and from the skin can follow an unpredictable course, draining to multiple lymph nodes (Uren, et al. 2003). To address the lymphatic drainage of the drug from the SC site of administration by lymphatic capillaries, and to quantify the volume and variability in vessel volume, we elected to parameterize this compartment as a fraction of injection volume. The rationale for this approach is predicated on the assumptions that (1) there are multiple afferent vessels draining the site and (2) the drainage volume would increase as the volume of injection increases due to spreading within the interstitial space (Porter, et al. 2001). Precedent for this position is based on comparisons of high pressure SC auto injector and manual SC injection of recombinant human growth hormone, where increased pressure from the auto injector resulted in a higher and earlier CPT peak compared to a manual syringe, which was attributed to greater spread of injected material in the SC space (Verhagen, et al. 1995).

Other PBPK models with injection depots have employed varying assumptions for lymphatic drainage. Gill et.al. parameterized the SC depot as a fixed volume and lymph flow estimated from literature reports on macromolecule disappearance from the SC site (Gill, et al. 2015). It’s unclear why the authors, however, elected to estimate lymph flow for each study rather than rely on a standardized the lymph flow rate for skin. Lymphatic flow is a physiologic process and expected to be driven by anthropometric characteristics variability; consequently, the methods outlined by Gill do not appear to readily lend themselves to population simulations. Moreover, an assumption of a fixed volume being attributed to the SC depot is not realistic as injection volume varies from compound to compound, and study to study based on drug physicochemical properties and tolerability to the injected drug concentration. Tegenge & Mitkus, in simulated intramuscular (IM) injections of squalene-containing compounds, parameterized the lymphatic drainage by taking the total lymph node volume and applying the fraction of lymph nodes assumed to drain directly from the IM depot space (Tegenge and Mitkus 2015; Tegenge and Mitkus 2013). This approach, however assumes the drainage occurs directly from injection site to a single node. However, patterns of lymphatic drainage from the skin are reported to vary substantially among individuals, even from the same area of the skin (Uren, et al. 2003).

Our model attempts to characterize local drainage by attributing a physical space representing the lymphatic capillary vessels the drug would journey through from the interstitial space to the lymph node, and is considered an additive volume to the lymphatic system as parameterized in the model proposed by Shah and Betts, (2012). Although it is well established that lymphatic vessels drain from the skin, to our knowledge, an experimentally obtained value for this volume has not be determined. Consequently, it is challenging to build a purely mechanistic model describing this drainage. Gill et. al. considered the disappearance of labeled IgG from the SC space as a means of estimating the transit of drug from the injection site to the vasculature, and others have used estimation procedures to fit a transit time parameter (Gill, et al. 2015; Shah and Betts 2012). However, a time parameter is not entirely mechanistic and cannot necessarily be relied upon for scaling up from preclinical species to human.

In contrast, parameterizing the LDC volume as a fraction of the injection volume represents an approach which assigns an anatomical volume to drug transit from the SC space to the lymph nodes relying on the skin lymph flow as a mechanistic driver of said transit. As this was the initial use of this methodology, no prior information was available to inform this value. Therefore we used mathematical estimation to optimize the value based on observed data obtained in the human population. Evaluation of the dose vs. exposure data suggested that the pharmacokinetic exposure was linear across the doses tested (unpublished data). Therefore we elected to perform the optimization based on all available observed data. However, as an informal sensitivity measure, we re-estimated Vfrac based on only a subset of the population which resulted in an indiscernible difference in the predicted vs. observed data (data not shown).

In the current model, the volume of the LDC was assumed to be dependent only on the injection volume, and independent of compound size. However, logically, spreading of drug particles within the SC space in theory could be influenced by the drug’s physicochemical characteristics such as fluid viscosity and inactive excipients. Therefore, the utility of our methodology requires qualification with compounds across a broad pharmacologic class and molecular weight, among other characteristics, to qualify this approach for more general use.

Another consideration in developing PBPK models for PK predictions of macromolecules after SC administration is the bioavailability after SC injection. Macromolecules are reported to vary in terms of their SC bioavailability, independent of molecular weight (Wang, et al. 2008; Richter, et al. 2012). The current compound was previously demonstrated to exhibit a relatively high SC bioavailability in primates at approximately 90 % (data not shown). Our model was largely able to predict the exposure in humans when scaled from primates, by assuming non-renal clearance processes are proportional to the volume of the space clearance occurs in. The predictive capacity of our model requires additional work, testing broader group of macromolecules with a wide range of SC bioavailability, to be confidently applied in an a priori setting for drugs with unknown SC bioavailability.

Based strictly on a percent change in AUC and Cmax, perturbation of the mean LDC does not appear as influential in terms of peak and overall exposure. In this scenario where the half-life was on average greater than 200 h based on non-compartmental analysis (data not shown), relying on the % change in AUC and Cmax is misleading, as inclusion of an LDC clearly was necessary to achieve the same profile shape as the observed data and resulted in a considerably smaller objective function. Inclusion of LDC only appears necessary to delay transit from the SC depot site immediately after administration. Following the initial transit through the lymphatics, drug mass that is not renally eliminated, recirculates to the organs. Drug mass that reaches the skin interstitial space that is not cleared by non-renal processes represents a relatively small proportion of the total circulating mass. Inclusion of a transit compartment draining from the full skin interstitial volume, parameterized identically to the drainage from the SC depot, consequently did not impact the model prediction (data not shown), and following the rule of parsimony, was excluded from the final model. However, incorporating lymphatic transit from the entire interstitial volume may have utility in other scenarios where multiple SC injection sites are being tested.

Despite the uncertainty in the mean and variability values included as input into the model for the lymph node compartment, the model was largely insensitive to the mean value of the lymph node. In contrast, the model exhibited the greatest sensitivity to the mean value for skin lymph flow likely as a consequence of the route of administration being SC. This is consistent with our previously developed model for predicting the mean time course of the same drug in humans from primates (Offman and Edginton 2015). We specifically observed a distinctly slower skin lymph flow was required relative to the current assumed range of 0.2–1 % of blood flow to an organ (Swartz 2001). Lymph flow is inherently difficult to measure in humans and has been assumed to scale from animal values. As a fraction of blood flow, it may not be reasonable to assume lymph flow represents a similar fraction of blood flow across all organs, and in fact Baxter reported a wide range of organ lymph flow values in the mouse, with skin lymph flow representing the lowest rate as an absolute value across all organs reported (Baxter, et al. 1994). Jones et. al. has stated that in spite of the wide range of reported lymph flows, many of the previously cited models fit the observed data well, and that this raises a fundamental question regarding model parameterization (Jones, et al. 2013). Jones goes on to state that approximately 0.07 % of fluid entering the interstitial space returns to the blood via the lymph when considering net fluid recirculation. It is interesting to note that in our previous work for the same compound (Offman and Edginton 2015), the optimized skin lymph flow was 0.1 %, very close to the 0.07 % of blood flow suggested by Jones et. al. More importantly, these results reinforce the argument that models being developed for IV administration cannot simply be applied to SC administration without consideration of specific lymphatic drainage from the SC depot site.

Assuming interindividual variability of skin lymph flow resulted in a wider prediction ribbon in the absorption and peak portions of the curve. The shape of the ribbon, particularly around Cmax, confirms the vital importance of this parameter in models intended to simulate the SC profile of a macromolecule and also further supports the previously stated position that skin lymph flow may represent a smaller fraction of blood flow than previously assumed.

Further evaluation of model sensitivity suggested clearance mechanisms are important for characterizing the interindividual variability of this pegylated peptide in a population. As previously stated, despite the large molecular mass, pegylated compounds can be renally eliminated in primates and humans albeit slowly, at a rate of approximately 0.1 % of GFR (Baumann, et al. 2014). As renal clearance accounted for approximately one third of total body clearance we were particularly interested in whether changes in renal function would result in a change in peak and overall exposure. Perturbation of FGFR, which in the current model represents a fraction of GFR, can be interpreted as a surrogate for a change in renal function and the results of the sensitivity analysis suggested that changes in renal function precipitate a change in AUC. Whether this percent change is sufficient to warrant a change in the recommended dose however, is a function of the drug’s safety profile and the relationship of exposure to safety.

## Conclusion

This is the first PBPK model incorporating lymphatic system anthropometric interindividual variability for the purposes of simulating macromolecule PK in a population. As a population generator, this model is capable of simulating a population of individuals across a wide range of body weight and heights for use with other compounds with lymphatic distribution and transport. A novel proposal of incorporating an anatomical space representing lymphatic drainage by lymphatic capillaries appears critical in characterizing the early time course following drug absorption and the consequence of excluding the compartment results in poor prediction of the observed data in the early portion of the CPT. Variability in this lymph transit compartment and skin lymph flow exhibit the greatest influence on model prediction as it pertains to the absorption phase of the CPT following SC administration, whereas blood volume and renal clearance exhibit the largest apparent effect on Cmax and elimination phases of the curve, respectively.

## Additional file

Additional file 1:
**Representative Mass Balance Equations.** (DOCX 22 kb)

## Abbreviations

FF: Renal plasma filtration fraction
FGFR: Renal clearance of the pegylated peptide conjugate expressed as a fraction of glomerular filtration rate
LO: Scaling factor organ blood flow is divided by to obtain lymph flow for all organs (except skin)
LS: Scaling factor skin blood flow is divided by to obtain skin lymph flow
NRCL: Non-renal clearance rate expressed in mL/hr/kg
σ_*i*_: Interstitial fluid reflection coefficient (See Fig. 1b)
σ_*v*_: Vascular reflection coefficient (varied by organ and optimized as per Offman and Edginton (2015))
σ_*sf*_: Vascular reflection coefficient scaling factor
Vfrac: Lymph drainage compartment volume expressed as a fraction of injection volume
LDC: Lymph drainage compartment
