# Supplementary material for: Population physiologically-based pharmacokinetic model incorporating lymphatic uptake for a subcutaneously administered pegylated peptide
Source: In Silico Pharmacol. 2016 Mar 1;4:3. doi: 10.1186/s40203-016-0018-5 (PMC4773320; doi:10.1186/s40203-016-0018-5)
Supplement: Additional file 1: — Representative Mass Balance Equations. (DOCX 22 kb) [file 40203_2016_18_MOESM1_ESM.docx]

Additional file 1: Representative Mass Balance Equations

Representative Mass Balance Equations for a Generic PBPK Model for SC Administration of a Pegylated Protein Conjugate with Renal and Non-Specific Clearance

Venous Plasma Circulation

$$V_{ven}*\frac{C_{ven}}{dt}=(Q_{heart}-L_{heart})*C_{pla,heart}+{(Q}_{adipose}-L_{adipose})*C_{pla,adipose}+\left( Q_{muscle}-L_{muscle} \right)*C_{pla,muscle}+\left( Q_{kidney}-L_{kidney} \right)*C_{pla,kidney}+\left( Q_{bone}-L_{bone} \right)*C_{pla,bone}+\left( Q_{skin}-L_{skin} \right)*C_{pla,skin}+\left( Q_{thymus}-L_{thymus} \right)*C_{pla,thymus}+\left( Q_{other}-L_{other} \right)*C_{pla,other}+\left[ \left( Q_{liver}-L_{liver} \right)+\left( Q_{smallintestines}-L_{smallintestines} \right)+\left( Q_{largeintestines}-L_{largeintestines} \right)+\left( Q_{spleen}-L_{spleen} \right)+\left( Q_{pancreas}-L_{pancreas} \right) \right]*C_{pla,liver}+L_{lymph}*C_{lymph}-Q_{lung}*C_{ven}-NRCL_{ven}*C_{ven}$$

Arterial Plasma Circulation

$$V_{art}*\frac{{dC}_{art}}{dt}={(Q}_{lung}-L_{lung})*C_{pla,lung}-(Q_{heart}+Q_{adipose}+Q_{muscle}+Q_{kidney}+Q_{bone}+Q_{thymus}+Q_{skin}+Q_{brain}+Q_{liver}+Q_{smallintestines}+Q_{largeintestines}+Q_{spleen}+Q_{pancreas}+Q_{other})*C_{art}-NRCL_{art}*C_{art}$$

Lymph

$$V_{lymph}*\frac{{dC}_{lymph}}{dt}=\sum\left( L_{organ}*\left( 1-\sigma_{i} \right)*C_{isf,organ} \right)+L_{depot}*C_{LTC}-L_{lymph}*C_{lymph}$$

Lung Vascular

$$V_{pla,lung}*\frac{dC_{pla,lung}}{dt}=Q_{lung}*C_{ven}-L_{lung}*\left( 1-\sigma_{sf}*\sigma_{v,lung} \right)*C_{pla,lung}-\left( Q_{lung}-L_{lung} \right)*C_{pla,lung}-NRCL_{pla,lung}*C_{pla,lung}$$

Lung Interstitial

$$V_{isf,lung}*\frac{dC_{isf,lung}}{dt}=L_{lung}*\left( 1-\sigma_{sf}*\sigma_{v,lung} \right)*C_{pla,lung}-L_{lung}*\left( 1-\sigma_{i} \right)*C_{isf,lung}-{NRCL}_{isf,lung}*C_{isf,lung}$$

Kidney Vascular

$$V_{pla,kidney}*\frac{dC_{pla,kidney}}{dt}=Q_{kidney}*C_{art}-L_{kidney}*\left( 1-\sigma_{sf}*\sigma_{v,kidney} \right)*C_{pla,kidney}-\left( Q_{kidney}-L_{kidney} \right)*C_{pla,kidney}-\mathrm{GFR}*\mathrm{FGFR}*C_{pla,kidney}-{NRCL}_{pla,kidney}*C_{pla,kidney}$$

Kidney Interstitial

$$V_{isf,kidney}*\frac{dC_{isf,kidney}}{dt}=L_{kidney}*(1-\sigma_{sf}*\sigma_{v,kidney})*C_{pla,kidney}-L_{kidney}*\left( 1-\sigma_{i} \right)*C_{isf,kidney}-NRCL_{isf,kidney}*C_{isf,kidney}$$

Skin Vascular

$$V_{pla,skin}*\frac{dC_{pla,skin}}{dt}=Q_{skin}*C_{art}-\left( L_{skin}+L_{depot} \right)*\left( 1-\sigma_{sf}*\sigma_{v,skin} \right)*C_{pla,skin}-\left( Q_{skin}-\left( L_{skin}+L_{depot} \right) \right)*C_{pla,skin}-{NRCL}_{pla,skin}{*C}_{pla,skin}$$

Skin Interstitial

$$V_{isf,skin}*\frac{dC_{isf,skin}}{dt}=L_{skin}*\left( 1-\sigma_{sf}*\sigma_{v,skin} \right)*C_{pla,skin}-L_{skin}*\left( 1-\sigma_{i} \right)*C_{isf,skin}-NRCL_{isf,skin}*C_{isf,skin}$$

Skin Subcutaneous Depot

$$V_{depot}*\frac{dC_{depot}}{dt}=L_{depot}*\left( 1-\sigma_{sf}*\sigma_{v,skin} \right)*C_{depot}-L_{depot}*\left( 1-\sigma_{i} \right)*C_{depot}-{NRCL}_{depot}*C_{depot}$$

Skin Depot Lymph Transit Compartment (LTC)

$$V_{LTC}*\frac{dC_{LTC}}{dt}=L_{depot}*\left( 1-\sigma_{i} \right)*C_{depot}-L_{depot}*$$

Liver Vascular

$$V_{pla,liver}*\frac{dC_{pla,liver}}{dt}=Q_{liver}*C_{art}+\left( Q_{smallintestines}-L_{smallintestines} \right)*C_{pla,smallintestines}+\left( Q_{largeintestines}-L_{largeintestines} \right)*C_{pla,largeintestines}+\left( Q_{spleen}-L_{spleen} \right)*C_{pla,spleen}+\left( Q_{pancreas}-L_{pancreas} \right)*C_{pla,pancreas}-L_{liver}*\left( 1-\sigma_{v,sf}*\sigma_{v,liver} \right)*C_{pla,liver}-\left[ \left( Q_{liver}-L_{liver} \right)+\left( Q_{smallintestines}-L_{smallintestines} \right)+\left( Q_{largeintestines}-L_{largeintestines} \right)+\left( Q_{spleen}-L_{spleen} \right)+\left( Q_{pancreas}-L_{pancreas} \right) \right]*C_{pla,liver}-{NRCL}_{pla,liver}*C_{pla,liver}$$

Liver Interstitial

$$V_{isf,liver}*\frac{dC_{isf,liver}}{dt}=L_{liver}*\left( 1-\sigma_{sf}*\sigma_{v,liver} \right)*C_{pla,liver}-L_{liver}*\left( 1-\sigma_{i} \right)*C_{isf,liver}-{NRCL}_{isf,liver}*C_{isf,liver}$$

Remaining Organs Vascular

$$V_{pla,organ}*\frac{dC_{pla,organ}}{dt}=Q_{organ}*C_{art}-L_{organ}*\left( 1-\sigma_{sf}*\sigma_{v,organ} \right)*C_{pla,organ}-\left( Q_{organ}-L_{organ} \right)*C_{pla,organ}-NRCL_{pla,organ}*C_{pla,organ}$$

Remaining Organs Interstitial

$$V_{isf,organ}*\frac{dC_{isf,organ}}{dt}=L_{organ}*\left( 1-\sigma_{sf}*\sigma_{v,organ} \right)*C_{pla,organ}-L_{organ}*\left( 1-\sigma_{i} \right)*C_{isf,organ}-{NRCL}_{isf,organ}*C_{isf,organ}$$

Notation

FGFR: Fraction of glomerular filtration rate attributed to renal clearance

GFR: Glomerular filtration rate

*isf* (subscripted): Interstitial fluid

*L*: Lymph flow

*NRCL*: Non-Renal clearance

*pla* (subscripted): Plasma

*Q*: Blood Flow

Vfrac: Fraction of injection volume attributed to the lymph transit vessels

*V_art_, V_ven_*: Volume of the arterial and venous plasma space

*V_lymph_*: Volume of the lymph node space

*V_isf,organ_*: Volume of the organ interstitial space

*V_pla,organ_*: Volume of the organ plasma vascular space

*σ_sf_*: Vascular reflection coefficient, scaling factor

*σ_v,organ_*: Organ vascular reflection coefficient

σ*_i_*: Interstitial fluid reflection coefficient
